# Emotional Valence from Facial Expression as an Experience Audit Tool: An Empirical Study in the Context of Opera Performance

**DOI:** 10.3390/s23052688

**Published:** 2023-03-01

**Authors:** Silvia Ceccacci, Andrea Generosi, Luca Giraldi, Maura Mengoni

**Affiliations:** 1 Department of Education, Cultural Heritage and Tourism, Università degli Studi di Macerata, P.le Luigi Bertelli 1, 62100 Macerata, Italy; 2 Department of Industrial Engineering and Mathematical Sciences, Università Politecnica delle Marche, Via Brecce Bianche 12, 60131 Ancona, Italy; 3 Emoj srl, Via Ferruccio Fioretti 10/B, 60131 Ancona, Italy

**Keywords:** customer experience, customer satisfaction, emotion recognition, artificial intelligence, facial expression recognition

## Abstract

This paper aims to explore the potential offered by emotion recognition systems to provide a feasible response to the growing need for audience understanding and development in the field of arts organizations. Through an empirical study, it was investigated whether the emotional valence measured on the audience through an emotion recognition system based on facial expression analysis can be used with an experience audit to: (1) support the understanding of the emotional responses of customers toward any clue that characterizes a staged performance; and (2) systematically investigate the customer’s overall experience in terms of their overall satisfaction. The study was carried out in the context of opera live shows in the open-air neoclassical theater Arena Sferisterio in Macerata, during 11 opera performances. A total of 132 spectators were involved. Both the emotional valence provided by the considered emotion recognition system and the quantitative data related to customers’ satisfaction, collected through a survey, were considered. Results suggest how collected data can be useful for the artistic director to estimate the audience’s overall level of satisfaction and make choices about the specific characteristics of the performance, and that emotional valence measured on the audience during the show can be useful to predict overall customer satisfaction, as measured using traditional self-report methods.

## 1. Introduction

Over the past decade, performing arts organizations have faced rising costs, severe funding cuts both from government and philanthropic sources [1], and a reduction in audience sizes due to increasing competition in an economy referred to as the “experience economy” [2]. Moreover, the downsizing of the number of spectators will likely characterize the next few years following the application of distancing measures made necessary by the COVID-19 pandemic, making programming activities more critical as the profit margins will suffer understandable decreases. Hence, art organizations need new tools to support audience understanding and development. Performing arts (e.g., opera) are complex, experiential, and emotionally charged [3]—much more than other kinds of services. They arouse cognitive and emotional experiences that affect the level of satisfaction of the audience so much that audience experience represents one of the most significant outputs of an arts organization [4]. To enhance audience understanding and development, performing arts organizations have become increasingly interested in how audiences make sense of their experiences [4]. First and foremost, they need to learn about customer satisfaction. To meet the audience’s desires, they should focus on the determinants of customer satisfaction and learn about what people like and dislike, or find attractive and unappealing [5]. While traditional marketing approaches in the performing arts have mainly focused on the analysis of attendance, its antecedents, and goals, neglecting the effects of attendance on spectators [5,6], in the case of opera performances, marketing efforts should concentrate on the core service quality rather than the peripheral service quality, and the opera organization should focus on the emotional satisfaction of their spectators [6]. Much research has recognized the key role of emotions during the entire customer experience (CX), and in marketing research, it is well-known how customer emotional responses affect cognitive judgments related to quality service, customer satisfaction, and behavioral intentions [4,7,8]. According to [9], emotions are discrete, automatic responses to universally shared, culture-specific, and individual-specific events. Understanding customers’ emotions toward any clue that characterizes an opera performance could be useful for the arts organizations to comprehend the audience’s mood, determine the level of the perceived quality, and hence evaluate if the choices made in the programming of the entire opera season respond to the audience’s expectation. These analyses can be a valid starting point for planning the next season, which, from the point of view of sales, could count on a core of customers satisfied with the quality delivered and the emotion developed during the performances. There are several methods for measuring emotions [10,11]. The majority of the studies on emotions during the CX typically consider only retrospective self-report measures [6,12,13]. They mainly focus on emotional appraisal rather than on effective customers’ emotions, not allowing the dynamic nature of emotions during CX to be captured and measured in real-time. Moreover, the collected measures can be affected by a bias due to subjective judgments [14]. Only a few studies considered neurophysiological measures to collect the dynamic nature of emotions during CX, e.g., [7,15]. However, they require the use of wearable sensors (i.e., ECG, GSR) to be collected. Given the intrusiveness of such devices, the results are strongly limited by the experimental conditions and the low number of users involved. Moreover, such technologies cannot be easily applied in uncontrolled settings, but only in a traditional laboratory setting. In the last few years, based on the Facial Action Coding System (FACS) developed by Ekman and Friesen [16], new emotion recognition systems based on facial expression analysis have been introduced. Such technology is capable of correlating the position of specific facial landmarks with Ekman’s “Big Six” [16,17], so that they can be easily used to monitor spectators during performances without being intrusive. Today, cameras are everywhere, so much so that they have become an integral part of our daily lives. We are all so accustomed to the presence of video surveillance cameras that we no longer care about them. This paper aims to explore the potential offered by emotion recognition systems to provide a feasible response to the growing need for audience understanding and development in the field of arts organizations. An empirical study investigates whether the emotional valence measured on the audience through an emotion recognition system based on facial expression analysis can be used as an experience audit, providing an alternative to the traditional self-report methods [18]. This method would have a dual purpose:

(1) Supporting the understanding of the emotional responses of customers toward any clue that characterizes a staged performance.
(2) Systematically investigating the customer’s overall experience in terms of overall customer satisfaction.

The rest of the paper is organized as follows: After an overview of the main techniques to measure customers’ emotions in uncontrolled settings such as an open-air opera festival, an emotion recognition system based on the combined use of deep learning and computer vision is presented. Section 3 illustrates the proposed method to assess the audience’s emotional behavior, while Section 4 describes the experiment conducted to evaluate the feasibility of the system and its ability to predict audience satisfaction and discusses the achieved qualitative results.

## 2. Research Background

### 2.1. Measuring Customers’ Emotions

For decades, service research has measured customers’ emotions by using self-report methods. This approach has been strongly supported by the assumption that customers’ emotions are strong mediators of overall customer satisfaction [3,19,20] and future behavioral intention, i.e., intention to repurchase or to return [21,22]. However, although this approach offers undoubted advantages (e.g., it is generally cheap, does not require special equipment, allows fast data collection, and enables the reach of wider samples [12,23]), it has evidenced several limitations. Many scholars argued that self-reported measures can be biased, e.g., by people’s ability to remember or explain [24], by the fact that people might be unwilling to answer [25], by people’s intention to answer in a socially desirable way [26,27], or by their tendency to give overly positive or negative responses [28]. Another cause of bias is related to the fact that self-reports do not allow in-the-moment measurement of a CX [29,30]. There is usually a significant time lag between the time that people experience emotions and the reporting time [15]. Some scholars have argued that reported self-measures can provide more of a description of people’s retrospective feelings about emotions (i.e., cognitive appraisals) than on emotions themselves [31,32] and may result in recall bias [33] or generate inconsistency [34]. Alternatively, several technologies nowadays available allow capturing, in an automatic way through neurophysiological measures, the dynamic nature of emotions during the CX. They potentially resembled collecting both people’s conscious and subconscious emotional responses objectively [7,35]. Such techniques differ mainly at an intrusiveness level, and most refer to three research areas: facial expression analysis, speech analysis, and biofeedback measures [15,36]. Several of these systems are commercially available today. Currently, there exist different emotion recognition software tools: Noldus’s FaceReader [37], iMotions’s AFFDEX module [38,39,40], and Visage Face Analysis [41] are just some examples. Today, a widely used approach in face-coding software delivery is to also make APIs available with cloud services. Google, Microsoft, and Amazon have been moving in this direction with their platforms for different years [42,43,44]. All these tools ensure good reliability per se. However, their effective applicability in a real context strongly depends on their reconfigurability and the characteristics of the dataset adopted for their training [45]. Despite being generally more accurate, the use of biofeedback tools, e.g., electroencephalography (EEG), electrocardiography, and sensors to measure galvanic skin response (GSR), can strongly affect the subjects’ behavior, especially their spontaneity, and consequently the emotions they experience during the observation [36,46]. In addition, these tools are expensive and time-consuming, as they require relatively expensive equipment (i.e., sensors) that must be placed appropriately on people’s bodies [25]. For this reason, in the last few years, many efforts have been made to improve the accuracy of non-intrusive emotion recognition systems based on speech and facial coding analysis. However, the effectiveness in general of systems based only on speech analysis still appears to be low, compared with systems based on facial expressions [47], as the acoustic parameters of speech can be influenced by many factors that cannot be directly correlated with emotional behavior. Most of the facial expression recognition systems available today make use of Deep Neural Networks [48], especially Convolutional Neural Networks (CNN), taking pictures of human faces as input and providing a prediction of the relative Ekman’s basic emotions (i.e., happiness, surprise, sadness, anger, disgust, and fear) [17], according to the trained model. Despite the potential of these new emerging technologies, very few studies in marketing service research have examined so far customer emotions as an ongoing and dynamic process by using neurophysiological measures, e.g., [7,15]. In addition, because these studies considered emotion recognition systems based on ECG and GSR measurements, they were conducted in a laboratory setting or involved a tiny number of users, so their results are limited and difficult to generalize. To the best of our knowledge, there are no studies yet that have evaluated the possibility of using a non-invasive emotion recognition system to estimate overall customer satisfaction based on facial expression analysis to analyze the emotional behavior of people during a show.

### 2.2. The Considered Emotion Recognition System

The emotion recognition system adopted in this study represents a state-of-the-art system for emotion recognition based on the analysis of facial expressions. Such software implements a combination of facial recognition and gaze-tracking technologies based on artificial intelligence algorithms, as described in detail in [45] and [49]. It enables the monitoring of the emotional state of people shot by a camera and their corresponding level of interest and involvement. Moreover, it exploits the solution described in [50] to recognize people’s age and gender. The adopted CNNs were trained with very large datasets retrieved in different application contexts (see [45]). This ensures system reliability even in the case of people with different facial appearances (e.g., children, adults, elderly, men, women, using cosmetics, wearing glasses, with beards of facial hair, with wrinkles), and high system performance also in the case of “wild” environments. It can detect up to fifteen people with a single camera, and it can detect even more people at once by combining many cameras. The only limit for monitoring many people simultaneously is the power of the computer that runs the system, while a normal PC or smartphone can run the single-person detection version. The software is not heavy, despite the fact that the inner CPU processes all the data and no information goes to the external server. Moreover, as the system works in real-time because images are suddenly processed and then erased without any association between the person’s name or ID and his/her face, this avoids privacy issues, according to the GDPR 2016/679 about privacy. Emotion identification is based on the work of psychologist Paul Ekman [17]. The choice to use Ekman’s basic emotions is motivated by their validity for all types of people from different cultures and different ethnic groups. However, the person’s emotional state at a specific time will be the result of a blend of these universal basic emotions. Although a discrete partitioning of emotions is a useful simplification to work with, the actual perception of feelings is complex and co-continuous. For this reason, Russel developed the Circumplex model [51], which provided a representation on a two-dimensional plane formed by Valence and Arousal, and later evolved into Mehrabian’s PAD (Pleasure-Arousal-Dominance) system [52]. Therefore, this software also provides an estimation of the emotional valence through a weighted average. It provides two indexes that offer a more complete picture of the emotional state of a user: valence and engagement. The valence allows differentiating between pleasant and unpleasant emotions, then provides a trend related to the positivity or negativity of the emotion felt in real-time, on a scale from −100 to +100. In general, happiness and surprise are considered positive emotions, while anger, sadness, disgust, and fear are considered negative emotions. On this scale, neutrality will have the value 0. The engagement provides a simple scale given by the intensity of the emotion felt, regardless of whether it may be negative or positive, providing a value from 0 (total absence of emotion) to 100 (highest level of emotion felt). The system also provides an estimation of people’s attention based on the method reported in [53]. It correlates the angle of rotation of the face with a point of interest for a certain time interval and the level of attention that the person feels for the point of interest itself. The software will consider interesting a person with a face rotation angle within a range that varies from ±10 to ±25 degrees depending on the context and the axis considered (*x* or *y*). The concrete applications of this technology are countless, both in the physical and digital worlds. Compared with the other tools and methods for emotional recognition and analysis, this system presents many advantages, such as:

- Non-invasive solutions that integrate several technologies;
- A modular architecture of technologies that let each module work as a stand-alone tool so that the functionality of the system is not compromised when modules are missing;
- Web-based user interface, easily accessible remotely yet protected by security protocols. Thus, the user can access data in a cloud-based environment;
- Emotion recognition technology inserted in a customer experience context.

Several studies have been carried out to determine the impact of such a tool on customer service management in many fields, ranging from automotive [54] to retail [46], e-commerce [49], e-learning [55], and museums [56]. They demonstrated that this technology represents an effective and non-invasive tool for continuous and real-time customer emotion monitoring in the wild. In digital contexts, it may represent a useful tool in the case of e-commerce to improve the conversion rate because it allows knowing precisely which elements of a web page are more effective in attracting a user’s attention and the emotions every detail can arouse. Consequently, it makes it possible to evaluate every part of the text, images, videos, calls to action, links, and other elements that can contribute to increasing the sales of an e-shop. Moreover, it may enable the assessment of teachers’ abilities based on students’ levels of engagement and attention and the changes in their emotional state during the lessons.

## 3. Materials and Methods

### 3.1. Research Design

This study aims to evaluate the usefulness of the proposed system to analyze, at a qualitative level, the experience behaviors expressed by the audience during the opera staging. To this end, starting with the assumption that “customer satisfaction can be considered essentially the culmination of a series of customer experiences or (…) the net result of the good ones minus the bad ones” [57], this study focuses on the analysis of the collected emotional valence, determined as the balance between the pleasant (i.e., joy and surprise) and the negative emotions (i.e., anger, fear, disgust, sadness) manifested by the audience alongside the show. It aims to understand whether the analysis of such data can provide precise indications, as an experience audit, by systematically investigating the customer’s overall experience during the performance that takes place on the stage and elucidating the emotional responses of customers toward any clue. Moreover, this study aims to assess whether the analysis of emotional valence manifested by the audience during the show can be useful to predict overall customer satisfaction, as measured using traditional self-report methods. To this end, the proposed system was applied in the context of opera live shows in the open-air neoclassic theater Arena Sferisterio, in Macerata (Italy), to collect neurophysiological measures and analyze the dynamics of the audience’s emotional behavior during the representations of the Macerata Opera Festival. Quantitative data aimed at investigating the level of customer satisfaction was collected using a proper questionnaire, as described below.

### 3.2. Emotional Response Monitoring

**Figure 1:**
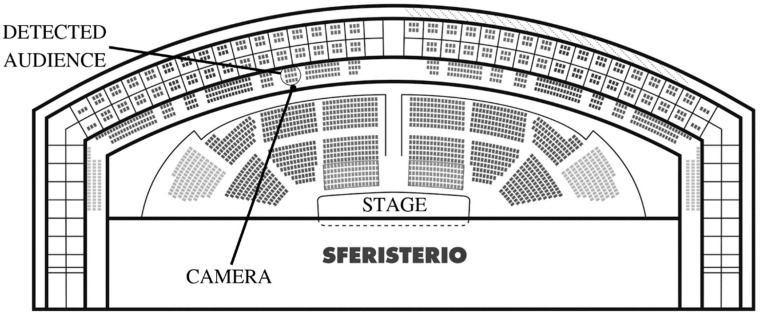
Map of the Sferisterio Arena. Area of the central sector subjected to monitoring.

**Figure 2:**
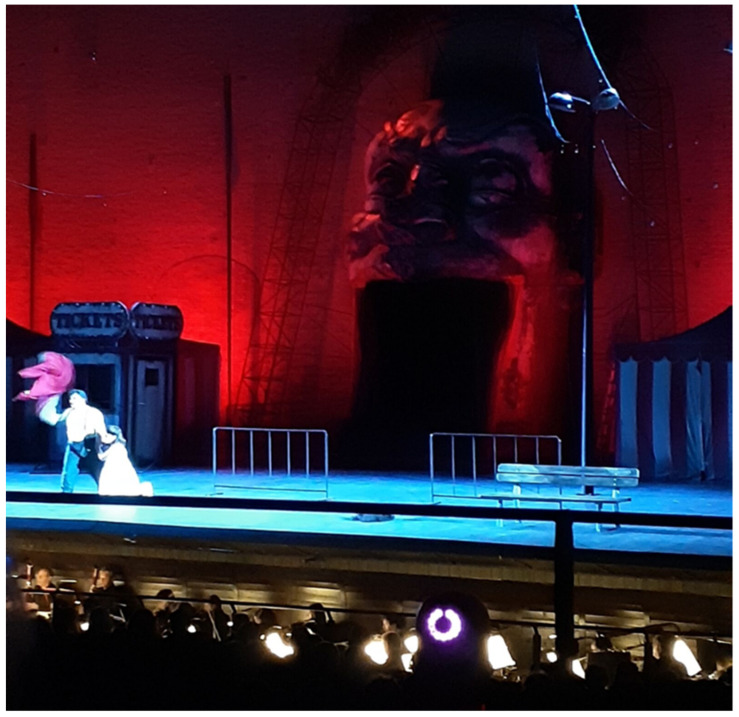
The IP Camera used to record the viewers’ faces during the performance.

The proposed emotion recognition system was used to monitor the audience during 11 opera performances. This monitoring was specifically conducted during 4 performances of Carmen, 3 performances of Macbeth, and 4 performances of Rigoletto. During each performance, 12 spectators were selected for the study whose profiles fit with the target audience. They were asked to sit in the central sector (Figure 1), where an infrared camera was positioned to track their levels of attention and emotions during the show (Figure 2). The monitoring took place between nine o’clock and midnight, with the stage lights representing, along with the stars, the only source of light during the performances. Under these conditions, it was therefore necessary to use an infrared camera, positioned on one of the railings in front of the selected audience, and connected to a local laptop via wifi connection. Specifically, the technical configuration adopted was as follows:

A Foscam R2 1080p infrared IP Camera;

An Asus VivoMini UN62 with Intel Core i5-4210U processor, 4 GB RAM.

Using the infrared mode, frame acquisition was conducted in grayscale; this did not cause any particular problems for the aforementioned CNN [45], trained with gray-scale converted images, although a slight decrease in accuracy was noted in some moments, likely due to the lack of definition in the acquisition of facial features when the infrared LEDs were not sufficient to illuminate the faces of the viewers. Other cameras were used to record performances and allow the acquired data to be temporally related to the events that occurred at the scene. The predictions provided by the system regard the probability from 0 to 100 that a photo belongs to one of Ekman’s emotions; these percentages, along with the timestamp, were saved in CSV format on the laptop connected to the camera, which is responsible for both neural network processing and data storage. The system, echoing the approach provided in [58], uses an algorithm to uniquely identify a subject as long as he/she remains in a spatial surround within the frame taken by the camera so that emotional data can be traced back to different subjects. In this context, in which the subjects were immobile, this system performed particularly well.

### 3.3. Self-Reported Measures

The qualitative analysis conducted with the emotion recognition system to detect the emotions of the audience was flanked by a quantitative survey aimed at investigating the level of customer satisfaction reported by spectators.

Based on the model of customer satisfaction in opera proposed in [6], we considered customer satisfaction to consist of (1) customers’ cognitive perception of the core service (i.e., the perceived artistic performance quality), (3) the perceived quality of the peripheral services, distinguishing between dimensions operating during the performance and dimensions operating before and after the performance. Consequently, we defined a questionnaire, adapting the one proposed in Table 1 [6]. The questionnaire was administered at the end of the performance. Participants were asked to answer by using a 1–5 Likert scale with verbal anchors, with 1 indicating the lowest quality (i.e., ‘‘strongly disagree’’) and 5 indicating the greatest quality (i.e., “strongly agree’’).

### 3.4. Participant Recruitment

The audience sample had been selected to correspond to the five Sferisterio buyer personas (Table 2), elaborated after a deep analysis of data derived from surveys conducted by the “Sferisterio Association” in the last two seasons of the Macerata Opera Festival.

### 3.5. Data Collection

The actual experiment consisted of monitoring participants during an opera performance through an emotion recognition system and asking them to answer a survey at the end of the performance. A total of 132 people were involved. Participation was encouraged by the offer of free tickets. Participants were informed about the objectives of the study, the types of data, and how they would be collected and processed, and they signed an informed consent form.

## 4. Result and Discussion

**Figure 3:**
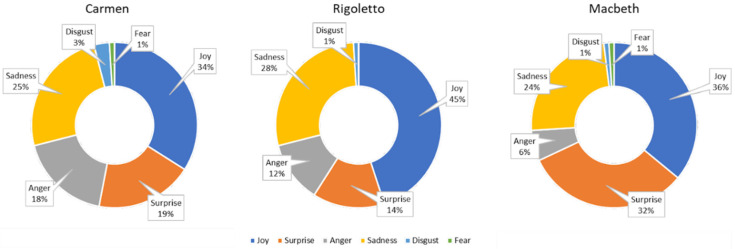
Ekman’s emotions registered during all the monitored performances of the three operas.

**Figure 4:**
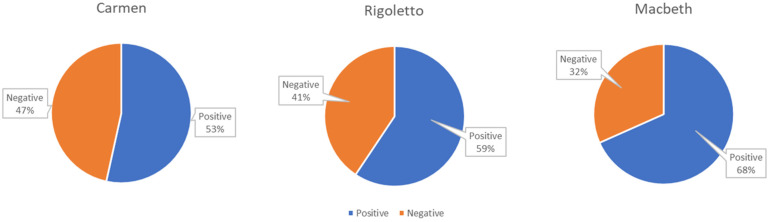
Emotional valence registered during all the monitored performances of the three operas.

Figure 3 and Figure 4 report the overall results of the emotional analysis carried out through the emotion recognition system on the audience of the Macerata Opera Festival for all the monitored Carmen, Rigoletto, and Macbeth performances. The system also allows for capturing audience engagement. This information is used to filter out only the emotional data that refers to times when audience members were engaged with the performance and were considered for analysis. In particular, the pie graphs show the valence of the emotions manifested overall by the audiences during all the monitored performances of Carmen, Macbeth, and Rigoletto, respectively. A negative valence can be determined by a general state of boredom or annoyance and frustration (i.e., sadness and anger) that derives from low interest in the show or indicates unpleasant sensations (i.e., disgust and fear).

**Figure 5:**
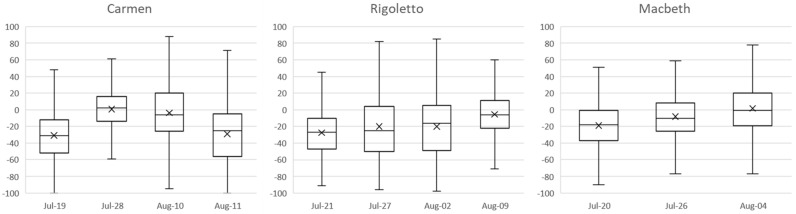
Emotional valence registered during each performance.

As can be observed, spectators who attended the performances of Carmen generally showed a higher level of negative emotions compared with those who attended Rigoletto or Macbeth. The performances of Macbeth were the ones that elicited the most positive emotions. For each spectator, the mean emotional valence *V_s* experienced during the performance was computed according to the following equation:

(1) $$V\_s=\frac{\sum_{1}^{n}v_{i}}{n}$$

where $v_{i}$ is the emotional value predicted from the *i*th frame and *n* is the total number of video frames analyzed for the considered spectator during the spectacle. This equation represents the average emotion value throughout the recording, distributed over all frames belonging to the recording for each subject. This calculation was required by the behavior held by the emotion analysis software, which, during the analysis of the recordings, processed the frames frame-by-frame and returned the emotional values for each of these frames. Figure 5 reports the level of mean emotional valence registered during each performance.

In the case of a complex performance such as a lyric opera performed in an open space at night, many factors can contribute to arousing emotions in the audience, such as the plot, the acting and direction, the scenography elements, the performances of the orchestra, the chorus, and the principal singers, the popularity of the main arias, the musical characteristics (e.g., timbre, melody, harmony, rhythm, intensity), and so on. By comparing the results of the analysis on the audience of the different representations, it is possible not only to indicate which opera the audience mostly appreciated but also to understand the consequences on the lived experience due to the differences among the representations of the same opera.

**Figure 6:**
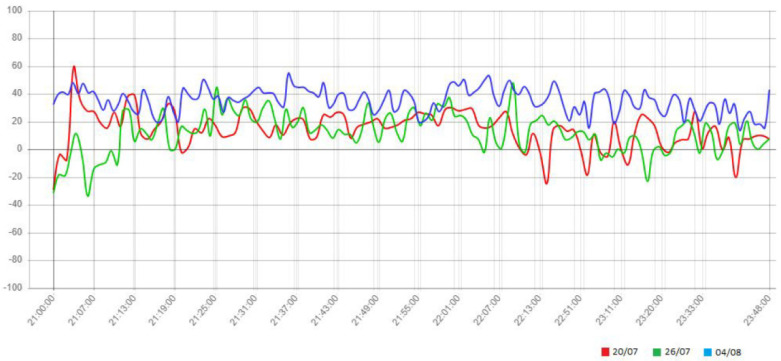
Trend of emotional valence during the three performances of Macbeth.

For example, by comparing the trend of the emotional valence registered during the three representations of Macbeth (Figure 6), it can be easily observed how the last representation has aroused emotions in the audience with more valence than the first two. A more accurate analysis of this trend can be useful to qualitatively estimate the level of appreciation related to the major events on the stage. To this end, based on the data timestamp, the emotional values collected from the audience can be referred to as the video recording of the correspondent show. In this way, it is possible to refer to each trend variation as an event happening on the stage. By analyzing the data, we noticed, for example, a positive peak in the correspondence of the following songs from Macbeth:

- Act I, scene I: Witches chorus Che faceste? dite su!;
- Act I, scene V: the cavatina of Lady Macbeth Vieni! t’affretta!/Come! Hurry!;
- Act II, scene V: brindisi Si colmi il calice/Fill up the cup;
- Act III, scene V: duet Vi trovo alfin!/I’ve found you at last;
- Act IV, scene I: the Scottish refugees chorus Patria oppressa/Downtrodden country;
- Act IV, scene I: aria of Macduff Ah, la paterna mano/Ah, the paternal hand;
- Act IV, scene IV: aria of Lady Macbeth Una macchia è qui tuttora/Yet here’s a spot.

**Figure 7:**
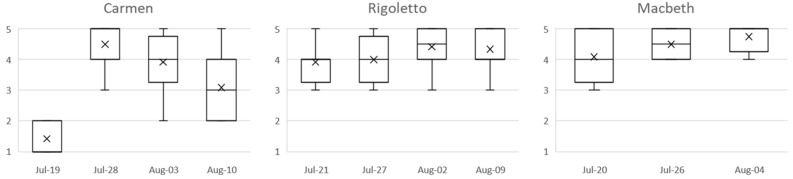
Customer satisfaction reported by the audience at the end of each performance.

The data collected also evidenced a significant increase in positive emotional reactions during the moments of applause; these highlight how the act of showing appreciation to the performers evokes powerful emotions in the spectators themselves. The link between applause and strong pleasant emotions should not be surprising; the results of several studies suggest that there is a link between clapping and happiness [59,60,61]. In this way, it is possible to gather information related to the audience’s response to the proposed show. Regarding the results of the customer satisfaction survey (Figure 7), scores related to the customer satisfaction (CS) and respective dimensions, i.e., the perceived artistic quality (AQ), the perceived peripheral service quality during the performance (SQ_D), and before and after it (SQ_B&A), were calculated by averaging the respective item values per participant. Internal consistency of all the scores was high (Cronbach’s on the pooled values: CS, α = 0.97; AQ, α = 0.91; SQD, α = 0.87; and SQBA, α = 0.89). The average of the ratings about CS collected by the spectators at the end of the various performances is shown in Figure 5.

Pearson product-moment correlation coefficients were computed to assess the relationship between the emotional valence and the levels of CS, AQ, SQD, and SQBA reported by the audiences. Results (Table 3) evidenced that there were positive correlations between valence and CS (r = 0.785, *p* < 0.01), valence and AQ (r = 0.621, *p* < 0.05), and valence and SQD (r = 0.618, *p* < 0.05). While there was no significant correlation between valence and SQBA, positive correlations were observed between CS and AQ (r = 0.960, *p* < 0.001) and between CS and SQD (r = 0.869, *p* < 0.01). There were no statistically significant correlations between the considered variables and SQBA.

Analyzing these results, it can be observed how the emotional valence measured during the performance can be useful in determining the overall level of satisfaction experienced by the spectators. Measured emotions seem to be directly elicited in the visitors by the stimuli they perceived during the performance (artistic quality, offered services, etc.). In contrast, results suggest that the quality of services offered before and during the performance (e.g., the kindness of the ushers, the ease of finding nearby parking, etc.) did not affect the emotions detected. These results suggest that the proposed emotion recognition system can serve as an experience audit tool to collect information regarding the level of satisfaction manifested by the audience during the performance. Monitoring the emotional valence would seem to be a possible alternative to collecting the subjective opinions of viewers through surveys.

## 5. Conclusions

This paper investigates whether the analysis of the dynamic nature of emotions manifested by the audience of performing arts (i.e., an opera performance) captured through a non-invasive emotion recognition system can support the understanding of the emotional satisfaction of customers toward any clue that characterizes the staged performance. Moreover, it suggests how the analysis of emotional valence manifested by the audience during the show can be useful to predict overall customer satisfaction, as measured using traditional self-report methods. The discussed results suggest that collected data related to audiences can be useful for the artistic director to estimate the audience’s overall level of satisfaction and better guide choices regarding the specific characteristics of the performance and casting, in order to improve the artistic offering. Furthermore, they seem to be useful in supporting the management of arts organizations in defining medium- and long-term strategies as well as marketing, both at a strategic and operational level. These results highlight the potential of a facial expression recognition system as an experience audit tool in the context of opera performances. However, future studies should be carried out to better understand its effectiveness even in the absence of integration with traditional research tools. Moreover, future studies should aim at investigating its effectiveness as well as its pros and cons in other performance art contexts. Once integrated into the marketing database activities, such information may constitute an important ingredient for the development of the CRM (Customer Relationship Management) system. In this sense, it will be possible to create offers that meet the qualitative and emotional expectations of customers, which are essential for building and nurturing a customer satisfaction path [62]. This can be read on an individual level, on a buyer persona (target) level, but also from an institutional point of view. In this sense, it is possible to develop the most appropriate positioning in light of the current market situations, taking also into account the competitive context and the characteristics of the arts organization. Market research has always proved to be fundamental in guiding the business strategies and practices of every type of actor and operator [63]. Some sectors have been using it for a long time, and others, such as the performing arts, have used it more recently. This is an excellent opportunity to start as innovators by making use of predictive tools related to technology, which in many ways can intercept more sincere and correct information in less time and with fewer problems related to privacy assurance. As for the truthfulness of the information, conventional methods for testing and predicting have always more often failed because they depend on consumers’ willingness and competency to describe how they feel when they are exposed to an advertisement, a product, or a service (opera in this case) [64]. The proposed emotion recognition tool, such as those that fall within the field of neuromarketing, provides cutting-edge methods for directly probing minds without requiring demanding cognitive or conscious participation.

## Figures and Tables

**Table 1:** Questionnaire for customer satisfaction in opera, adapted from [6].

Items	Answers
Customer Satisfaction	My expectations concerning the evening were met completely. All in all, the evening was an amazing experience for me. I am very disappointed with tonight. (R) This evening in the opera will leave a positive memory for a long time.
Perceived Artistic quality	All in all, I was convinced by the artistic quality of the performance. In my opinion, this was an artistic top performance. It would not be a loss, if this production was removed from the playing schedule soon. (R) I would expect this performance to receive the highest praise from the critics.
Perceived Peripheral service quality	**During the performance** The auditorium has very good acoustics. My seat was very comfortable. From my seat I had a very good view on the stage. During the performance, annoying distractions (e.g. noises) took place. (R) The auditorium’s temperature was very pleasant. **Before and after the performance** I was very pleased with the service of the house (Hospitality, cloak room, parking lots, etc.) The location where the audience lingers before and after the performance is pleasing and invites to stay. This theater’s ambience is very pleasant.

**Table 2:** Buyer Personas.

Buyer Personas of Macerata Opera Festival					
	Mauro Giacomini	Federico Antogirolami	Chiara Stortoni	Agnes Wurdistrow	Michele Auditore
Age	50	25	70	43	70
Gender	Male	Male	Female	Female	Male
Residence	Macerata	Province of Macerata	Milan	Germany	Ancona
Family	Married with 2 children.	Single with modest origins.	Husband retired, daughter independent	Single	Widower, lives with daughter and cat.
Education	Master’s degree.	Student at the Academy of Fine Arts of Macerata.	High school graduation	Master’s degree.	High school graduation
Job	Accountant	None	Entrepreneur and seamstress.	Lawyer.	Former bus driver, working in office, after losing the use of his legs in an accident
Salary	Very high	Very low (parents’ support).	Medium-high	High	Medium-low
Computer skills	Very high	Average	Low	Very high	Medium-high
Hobbies/Passions	Classical music, fine food and wine	Various art forms and music genres	Theater, opera, books (culture in general)	Classical and experimental theater	He runs a blog about music
Fears	His family does not share his passion for opera.	Opera seen as too "high” culturally therefore exclusive.	Used to attend La Scala theater, she thinks that the Sferisterio is addressed to an uneducated audience and that the productions are not of high quality.	Indecision about the type of travel, fear of postponement due to bad weather, fear of having to give up at the last minute for work, increasing prices over the years.	Living with disabilities in a bad way, fears that the Sferisterio is not adequately equipped to welcome people with his condition.
Needs	To improve time management between work and free time, to spend more time with his family.	Flexibility of schedule and low cost	To be informed about the world of opera and books.	Combine passion for theater with a need for social interaction.	To participate in more music events but needs to be accompanied.
What he is looking for Macerata Opera Festival:	Quality artistic productions and combination with wines.	Part-time work, useful both to earn money and to deepen your studies in the theatrical field.	An alternative to La Scala to attend an opera while on vacation i n the Marche.	Top tier works intended for an international audience.	Musical performances, easily accessible to people with disabilities (at reduced prices).
How to reach him:	Offline advertising in the city, email, and social channels.	Digital channels and collaboration with the academy.	Email and offline contact through the book club of which she is a member.	Email, social and regional tourism information channels.	All digital channels, agreements with organizations and associations for disabled people.
How to build loyalty:	Invitations to aperitifs, creation of works for the whole family.	Invitations to rehearsals, backstage tours and steep discounts.	Frequent and personalized updates, conventions with accommodation facilities and tourist activities at a regional level.	Increase the quality of the shows without raising prices and propose packages opera + tourism.	Priority to people in his condition, dedicated shows, and collaborations with non- profit organizations.

**Table 3:** Correlations (*n* = 132).

Scale	CS	AQ	SQD	SQBA
Valence	0.785 **	0.621 *	0.618 *	−0.167
CS		0.960 ***	0.689 **	0.053
AQ			0.887 ***	0.240
SQD				0.394

Valence, emotional valence; CS, customer satisfaction; AQ, perceived artistic quality; SQD, perceived peripheral service quality during the performance; SQBA, perceived peripheral service quality before and after the performance. *** *p* ≤ 0.001, ** *p* ≤ 0.01, * *p* ≤ 0.05.

## Data Availability

Not applicable.
